# A Magic Act in Causal Reasoning: Making Markov Violations Disappear

**DOI:** 10.3390/e27060548

**Published:** 2025-05-23

**Authors:** Bob Rehder

**Affiliations:** Psychology Department, New York University, 6 Washington Place, New York, NY 10012, USA; bob.rehder@nyu.edu

**Keywords:** causal reasoning, causal knowledge, Markov violations, rational process model, causal graphical models

## Abstract

A desirable property of any theory of causal reasoning is to explain not only why people make causal reasoning errors but also *when* they make them. The *mutation sampler* is a rational process model of human causal reasoning that yields normatively correct inferences when sufficient cognitive resources are available but introduces systematic errors when they are not. The mutation sampler has been shown to account for a number of causal reasoning errors, including *Markov violations*, the phenomenon in which human reasoners treat causally related variables as statistically dependent when they are normatively independent. A Markov violation arises, for example, when an individual reasoning about a causal chain X→Y→Z treats *X* as informative about the state of *Z* even when the state of *Y* is known. Recently, the mutation sampler was used to predict the existence of previously untested experimental conditions in which the *sign* of Markov violations would switch from positive to negative. Here, it was used to predict the existence of conditions in which Markov violations should *disappear* entirely. In fact, asking subjects to reason about a novel causal structure with nothing but *generative* causal relations (a cause makes its effect more likely) resulted in Markov violations in the usual positive direction. But simply describing one of four causal relations as *inhibitory* (the cause makes its effect less likely) resulted in the elimination of those violations. Theoretical model fitting confirmed how this novel result is predicted by the mutation sampler.

## 1. Introduction

Decades of research have asked: How do people draw causal inferences about the world around them? On the one hand, this research has revealed that people are often subtle and sophisticated causal reasoners: their causal inferences are sensitive to the direction of the causal arrow; they know that an intervention will affect the variable’s effects but not its cause; during learning they can control for alternative causes and are able to use temporal information to infer causal direction; and so on. Indeed, when it is available virtually all humans judgments are deeply influenced by causal knowledge [1,2,3,4,5,6]. Yet research has also shown that people commit systematic errors. So a challenge for theories of human causal reasoning is how to account for human ability to often draw sophisticated inferences while also explaining the common errors we make.

Recently, Davis and Rehder proposed a model called the *mutation sampler,* whose goal is to account for this phenomenon [7]. The mutation sampler is an instance of the species of models known as *rational process models*: models that are characterized by an algorithm for computing a normative answer but whose performance can be gracefully degraded when cognitive resources are scarce [8,9,10,11,12]. As a result, the mutation sampler yields predictions that are close-to-optimal (reflecting the normative algorithm) but that also exhibit systematic deviations from optimality (reflecting cognitive resource limitation). In fact, there is now a substantial body of evidence supporting the mutation sampler as a viable model of human causal reasoning [7,13,14,15].

An important step in the development of any new psychological theory is to identify novel situations for which the theory makes interesting and perhaps surprising predictions. In this article, I will first describe a robust causal reasoning error known as *Markov violations* and discuss how the mutation sampler accounts for such errors in a variety of past experimental conditions. On the basis of the explanation that the mutation sampler provides for Markov violations, I will then identify new experimental conditions for which such errors should, in fact, disappear. I will then present new experimental evidence testing those conditions.

## 2. The Mutation Sampler

**Figure 1:**
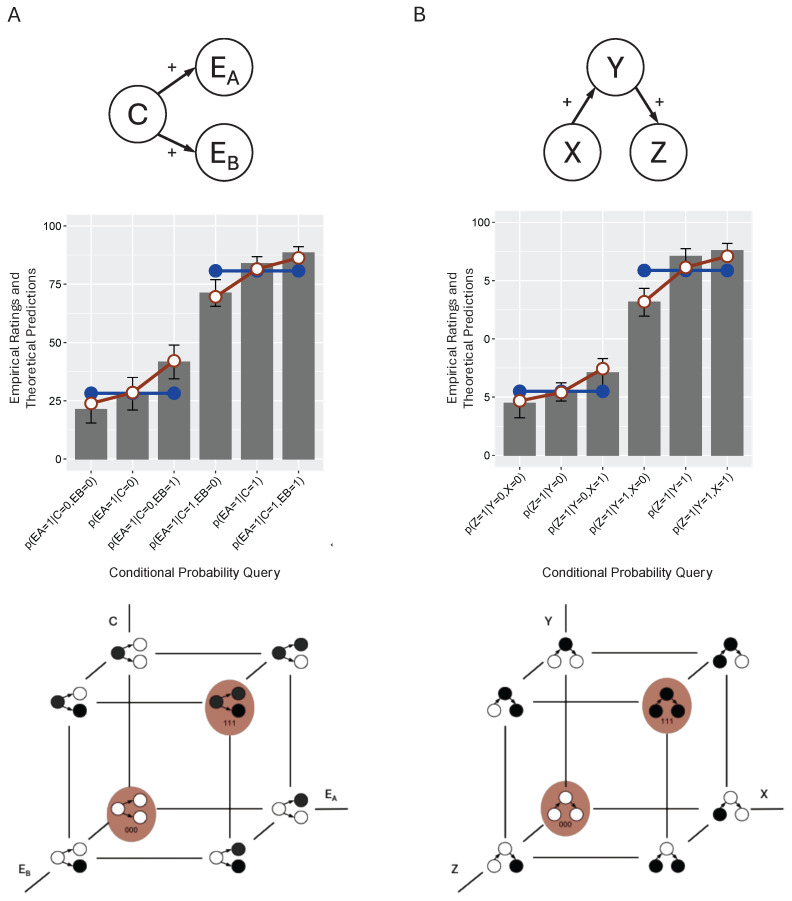
Empirical results from two conditions reported in one study of causal reasoning [15] and the associated theoretical model fits. Panel (**A**): The generative–generative condition of Experiment 1 that tested a common cause network. Panel (**B**): The generative–generative condition of Experiment 2 that tested a chain network. In one of three domains (economics, meteorology, or sociology), university undergraduates were taught a new theory consisting of hypothetical causal relations between binary variables and then asked to draw several causal inferences. Subsets of those inferences are shown on the x-axis of each graph, and the subjects’ average responses (recorded on a 0–100 scale) are the gray bars. The error bars are standard errors. The blue lines with closed plot points are the fits of the normative model, and the red lines with open plot points are those of the mutation sampler. The highlighted system states in the lattice at the bottom of each panel represent the prototypes at which the mutation sampler will commence sampling.

Figure 1A presents a typical scenario, in which experimental subjects are asked to draw causal inferences [15]. For example, in the domain of economics, meteorology, or sociology, university undergraduates were first taught novel causal knowledge that formed the *common cause* network shown in the top of the figure ($E_{A}\leftarrow C\to E_{B}$). In the domain of economics, some subjects were told that “Low interest rates cause small trade deficits” ($C\to E_{A}$) and also that “Low interest rates cause high retirement savings” ($C\to E_{B}$). They also received a short description of the mechanisms responsible for these causal relations. After learning this information, the subjects were asked to draw several causal inferences. For example, the subjects might be presented with an economy that had low interest rates and normal retirement savings and asked to predict whether it had small trade deficits; that is, they were asked to judge $p(E_{A}=1|C=1,E_{B}=0)$. The graph in Figure 1A presents subject ratings (on a scale of 0–100) for a number of conditional probability judgments.

The mutation sampler attempts to account for such judgments by assuming that a core component of causal reasoning is *sampling*; specifically, sampling over the states of the causal system being reasoned about. When its variables are binary, a common cause system consists of eight distinct states, as represented by the lattice at the bottom of Figure 1A. According to the mutation sampler, human reasoners draw causal inferences by first sampling over these states using Markov chain Monte Carlo (MCMC) methods; specifically, the Metropolis Hastings rule. As the number of samples grows large, this rule guarantees that the samples will approximate the network’s underlying joint distribution; that is, the probability that the system will be in each of its eight possible states. These approximated joint distributions can then be used to compute any causal inference (e.g., a conditional probability) that is needed.

As mentioned, without any additional assumptions the mutation sampler will simply approximate the causal network’s normative joint distribution. For example, suppose that we know that in a common cause network the probability of the cause *C* is represented by parameter $w_{c}$, that the strength of the two causal relationships is represented by $w_{C,E_{A}}$ and $w_{C,E_{B}}$, and that each effect can be caused by unspecified exogenous causes, causes not shown in the network, the strength of which is represented by $w_{E_{A}}$ and $w_{E_{B}}$. Also, suppose that the probability of an effect $E_{i}$ is given by a logistic function $p\left(E_{i}=1|C\right)=1/\left(1+exp\left(-\left(w_{CE_{i}}C+w_{E_{i}}\right)\right)\right)$, where the cause *C* is coded as 1 when present and −1 when absent. Given that the semantics of a common cause network allow us to write its joint distribution as $p\left(C,E_{A},E_{B}\right)=p\left(E_{A}|C\right)p\left(E_{B}|C\right)p\left(C\right)$, we can then compute the causal network’s joint distribution for any parameterization $w_{c}$, $w_{C,E_{A}}$, $w_{C,E_{B}}$, $w_{E_{A}}$, and $w_{E_{B}}$. This is the distribution that the mutation sampler’s MCMC samples will, when normalized, approximate.

But what makes the mutation sampler useful as a *psychological* model is that there are simple ways to degrade its performance so that its predictions diverge from the normative ones but potentially begin to mimic those of human reasoners. Two assumptions are needed. The first is that there is a limit on the cognitive resources that are available for sampling, so that only, say, a dozen or so samples are taken rather than the thousands or millions associated with MCMC methods when an accurate representation of a distribution is required. The second assumption is that sampling tends to commence at those causal network states that are perceived to be of high probability, states referred to by Davis and Rehder as *prototype states*. For example, in the case of the common cause network in Figure 1A the claim is that reasoners will carry out a superficial but low-cost analysis of the network and conclude that the states in which all variables are present (C=1, $E_{A}=1,E_{B}=1$, abbreviated “111”) or all are absent ($C=0,E_{A}=0,E_{B}=0$, “000”) are highly probable and, so, they are good places to start sampling. These states are highlighted in red in the lattice in Figure 1A. Note that the reasoner’s derivation of the prototype states is superficial in that it ignores all quantitative information (e.g., the causal strengths $w_{C,E_{A}}$ and $w_{C,E_{B}}$) and even the direction of the causal relations.

On the one hand, biasing where sampling begins has some justification. Davis and Rehder showed that, holding the number of samples constant, starting at high probability states leads to causal inferences that are generally more accurate for a wide variety of network parameterizations [7]. Yet it also introduces systematic errors. For example, a key property of causal Bayes nets is the *Markov condition*, which states that a variable is independent of its non-descendants conditioned on the states of its immediate parents [16]. Applied to the common cause network of Figure 1A, this means, for example, that the probability of $E_{A}$ should be unaffected by the state of $E_{B}$ when the state of *C* is known. In other words, *C* should *screen off* $E_{A}$ from $E_{B}$, such that $p\left(E_{A}=1|C,E_{B}=0\right)=p\left(E_{A}=1|C\right)=p\left(E_{A}=1|C,E_{B}=1\right)$ should hold for any value of *C*. Yet, conditional probability judgments derived from a distorted joint distribution—such as ones computed by the mutation sampler with a modest chain length and a bias in the starting point—will also be distorted, with the result that they may exhibit violations of the Markov condition.

The human judgments presented in Figure 1A are those that are relevant to assessing whether people honor the Markov condition when reasoning about a common cause scenario. On the left side of the graph are the three judgments regarding the probability of the effect $E_{A}$, given that the common cause is absent (C=0) as a function of the state of the other effect $E_{B}$: $p(E_{A}=1|C=0,E_{B}=0)$, $p(E_{A}=1|C=0)$, and $p(E_{A}=1|C=0,E_{B}=1)$. On the right are analogous judgments when *C* is present. The gray bars are the subjects’ responses to those queries on a 0–100 scale.

The blue lines represent the predictions of the normative causal graphical model (multiplied by 100), where the model’s parameters ($w_{C}$, $w_{C,E_{A}}$, $w_{C,E_{B}}$, etc.) are optimized to fit each subject’s complete set of judgments. That the blue lines are horizontal reflects the Markov condition: the probability of $E_{A}$ varies with *C* but not with the state of the other effect $E_{B}$. In contrast, the empirical results show that the human judgments violated the Markov violation: the probability of $E_{A}$ was generally lower when $E_{B}$ was absent (i.e., $p\left(E_{A}=1|C,E_{B}=0\right)<p\left(E_{A}=1|C\right)$) and generally higher when it was present ($p\left(E_{A}=1|C\right)<p\left(E_{A}=1\right|C,E_{B}=1$) both when C=0 and C=1.

The fitted predictions of the mutation sampler (red lines) illustrate how that model reproduces these violations. It does so because its assumption that sampling commences at one of the two prototypes combined with a limited number of samples introduces a distortion into the sampled representation of the causal model’s joint distribution and, so, the conditional probabilities derived from that joint distribution. This finding of Markov violations in the positive direction—$p(E_{A}=1|C,E_{B})$ is larger when $E_{B}$ is present versus absent—when testing common cause networks with generative relations has been observed in a large number of studies [17,18,19,20,21,22,23,24,25,26,27].

Tests of how people reason with other causal structures have corroborated the predictions of the mutation sampler. For example, the same study also tested subjects on a causal chain X→Y→Z, as shown in Figure 1B [15]. The Markov condition stipulates that a causal chain embodies another instance of conditional independence: it is now *X* and *Z* that should be independent, conditioned on *Y*. The graph in Figure 1B presents the subjects’ predictions regarding the presence *Z* given *Y*, varying whether *X* is absent, unknown, or present. The fits of the normative causal model (again, with parameters optimized to each subject’s ratings) shown by the blue horizontal lines illustrate the Markov condition: the state of *X* should have no effect on the estimated probability of *Z* when the state of *Y* is known. In contrast, the subjects committed Markov violations: *Z* was judged to be more likely when the supposedly screened-off *X* was present versus absent. Moreover, Figure 1B indicates that the fitted mutation sampler reproduces these judgments quite closely. Because its principles for determining prototypes are insensitive to causal direction, the lattice in Figure 1B shows that the mutation sampler assumes the same prototypes for a chain network as it did for a common cause network; namely, 000 and 111.

**Figure 2:**
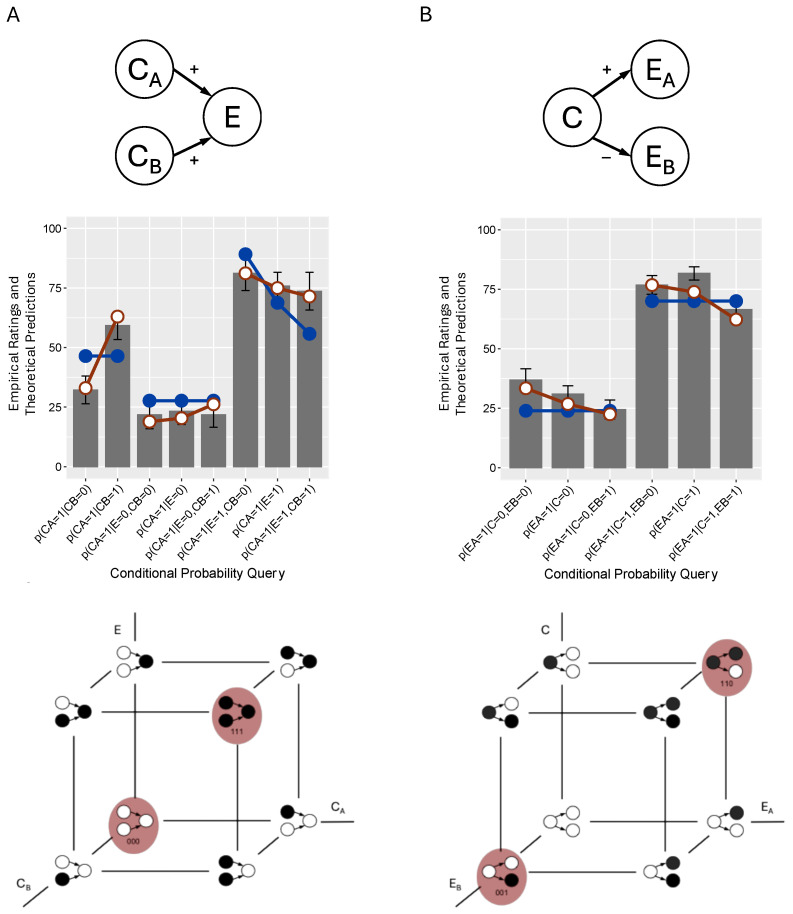
Empirical results from two additional conditions reported in [15]. Panel (**A**): The generative–generative condition of Experiment 3 that tested a common effect network. Panel (**B**): The generative–inhibitory condition of Experiment 1 that tested a common cause network with one inhibitory causal relation. The error bars are standard errors. The blue lines with closed plot points are the fits of the normative model, and the red lines with open plot points are those of the mutation sampler.

The study in [15] also tested a *common effect* network, in which the subjects reasoned with the network in Figure 2A, where $C_{A}$ and $C_{B}$ were each independent causes of *E*. Whereas when applied to common cause and chain networks the Markov condition stipulates that two variables become independent conditioned on a third, for a common effect network it stipulates that the two causes are *unconditionally* independent, such that, say, $p(C_{A}=1|C_{B}=0)$ = $p(C_{A}=1|C_{B}=1)$ should hold. The predictions of the normative causal model fitted to the empirical data in Figure 2A confirmed that $C_{A}$ and $C_{B}$ should be independent (horizontal blue line). In contrast, the subjects instead judged that $p(C_{A}=1|C_{B}=0)$ < $p(C_{A}=1|C_{B}=1)$. The fitted predictions of the mutation sampler indicate that it is able to reproduce this effect (assuming, again, prototypes of 000 and 111; see the lattice in Figure 2A).

Note that an important characteristic of common effect networks is *explaining away*, in which, when the effect *E* is known to be present the presence/absence of, say, $C_{A}$ results in the lowering/raising of the probability of $C_{B}$; that is, $p(C_{A}=1|E=1,C_{B}=0)$ > $p\left(C_{A}=1|E=1\right)>p\left(C_{A}=1|E=1,C_{B}=1\right)$ should hold. Explaining away is illustrated by the blue line in Figure 2A with the negative slope. The subjects, however, exhibited explaining away that was substantially weaker than predicted by the normative causal model. The mutation sampler’s prototypes reproduced this effect as well, as illustrated by the red line with a shallower negative slope. This result illustrates how the mutation sampler’s distorted joint probabilities affect the calculation of all conditional probabilities, not just those involved in the Markov condition. These results testing common effect structures—both the violations of independence and weak explaining away—have also been demonstrated in multiple studies [22,23,25,26,28,29,30].

Although the empirical tests reviewed so far all involved *generative* causal relations in which the cause increases the probability of its effect, ref. [15] also tested causal networks where one or more of the causal relations was described as *inhibitory*. For example, Figure 2B presents the results with a common cause network in which $C\to E_{A}$ and $C\to E_{B}$ were described as generative and inhibitory, respectively. Remarkably, whereas all previous demonstrations of Markov violations reported violations that were in the positive direction (e.g., $p(E_{A}=1|C,E_{B})$ is higher when $E_{B}$ was present versus absent), in this new condition they were *negative* ($p(E_{A}=1|C,E_{B})$ was lower when $E_{B}$ was present versus absent). The mutation sampler reproduces this result because, rather than assuming that the prototypes are 000 and 111, it assumes that they are 110 (*C* and $E_{A}$ both present, $E_{B}$ absent) or 001 (*C* and $E_{A}$ both absent, $E_{B}$ present), an assumption that is sensible given that the inhibitory relation between *C* and $E_{B}$ means that those variables should have opposite values. In fact, ref. [15] showed that Markov violations were negative for every causal network topology that involved a mixture of generative and inhibitory causal relations (and, intriguingly, they reverted to the positive direction when all causal relations were inhibitory) and that these results were accounted for by the mutation sampler.

## 3. The Magic Act: Eliminating Markov Violations

Given the robustness of Markov violations in psychological research—they have been observed in more than a dozen studies—an experimental result with both empirical and theoretical importance would be one where such errors *failed* to occur. The findings reviewed above demonstrated the value of the mutation sampler in being able to identify conditions in which the sign of Markov violations would switch from positive to negative. Here, the mutation sampler is used to identify conditions in which Markov violations should fail to appear altogether.

**Figure 3:**
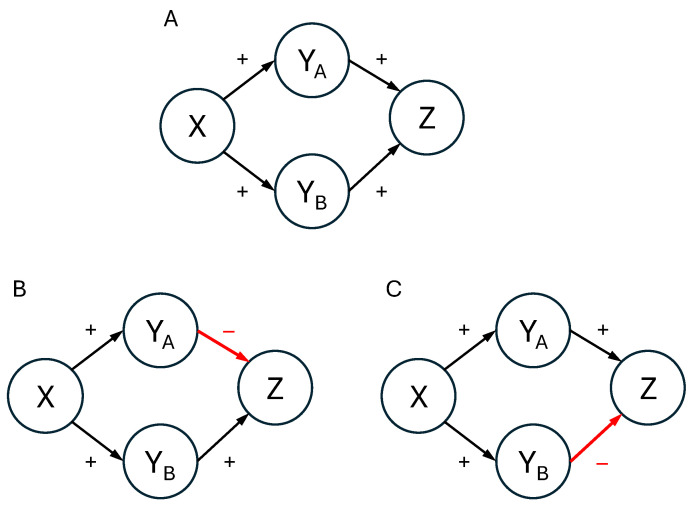
Three causal conditions tested in the current study. Panel (**A**): The Generative condition. Panel (**B**): The Y_A_-Inhibitory condition. Panel (**C**): The Y_B_-Inhibitory condition.

Consider the causal networks shown in Figure 3. In all three networks a variable *X* is the cause of both $Y_{A}$ and $Y_{B}$, which, in turn, are independent causes of *Z*. In Figure 3A, all four causal relationships are stipulated to be generative; that is, each cause makes its effect more likely. In contrast, in Figure 3B,C one of the four causal relationships is described as inhibitory instead. In Figure 3B the inhibitory relation is $Y_{A}\to Z$, whereas in Figure 3C it is $Y_{B}\to Z$.

Now, consider the independence properties of these networks. Firstly, there are no instances of unconditional independence. Any pair of variables that are directly causally related will exhibit dependence, and of those pairs that are not directly related, $Y_{A}$ and $Y_{B}$ are dependent on their common cause *X*, and *X* and *Z* are dependent because of the mediators $Y_{A}$ and $Y_{B}$. However, consider the situation where the state of *X* is known. As we have seen, a key prediction of causal Bayes nets is that in common cause networks (such as the $Y_{A}\leftarrow X\to Y_{B}$ subnetwork that appears in each panel of Figure 3) the two effects $Y_{A}$ and $Y_{B}$ are independent when conditioning on the cause *X*. Of course, $Y_{A}$ and $Y_{B}$ are also causally related to *Z*. However, we have also seen that a principle of common effect networks (such as the $Y_{A}\to Z\leftarrow Y_{B}$ subnetworks in Figure 3) is that the two causes $Y_{A}$ and $Y_{B}$ are independent so long as no information about *Z* is available. In summary, in all the networks in Figure 3 $Y_{A}$ and $Y_{B}$ should exhibit independence when conditioning on *X*.

The mutation sampler makes different predictions. Because it stipulates that sampling will begin at those network states that exhibit qualitative consistency with the causal relations, for the network with all generative relations in Figure 3A the prototypical states are either all four variables present (1111) or all four absent (0000). And the consequence of those prototypes should come as no surprise: subjects should commit positive Markov violations, just as they did for the networks in Figure 1 and Figure 2 with all generative relations. That is, instead of treating $Y_{A}$ and $Y_{B}$ as independent when conditioning on *X*, the mutation sampler predicts that they will judge that $p\left(Y_{A}=1|Y_{B}=0,X\right)>p\left(Y_{A}=1|X\right)>p\left(Y_{A}=1|Y_{B}=1,X\right)$ and $p\left(Y_{B}=1|Y_{A}=0,X\right)>p\left(Y_{B}=1|X\right)>p\left(Y_{B}=1|Y_{A}=1,X\right)$.

However, the mutation sampler’s predictions differ for the networks in Figure 3B,C. For these networks, the key question is: What are the prototypes? On the one hand, nothing in nature precludes real-world causal processes in which some variable *X* has a positive causal influence on a variable *Z* through one mediator but a negative influence through another. However, for a human reasoner who is carrying out only a superficial, qualitative analysis, the apparent contradiction that *X* makes *Z* both more and less likely means that these networks have no prototypes: that is, there are no settings of the four variables that are qualitatively consistent with all four causal relations. In such a case, Davis and Rehder speculated that there will be no bias in the starting point of the sampling chain, because there are no grounds for favoring one state over another: all are (qualitatively) (in)consistent with the causal relations [7]. And the consequence is that for the networks in Figure 3B,C Markov violations disappear.

These predictions were tested in the following experiment. The subjects were assigned to either a *Generative* condition, in which all causal relations were generative as in Figure 3A, or an *Inhibitory* condition, in which one causal relation was inhibitory. Within the Inhibitory condition, half of the subjects were told that $Y_{A}\to Z$ was inhibitory and the other half that $Y_{B}\to Z$ was inhibitory. Based on previous research, there was a strong expectation that positive Markov violations would be observed in the Generative condition. The main question asked here is whether the predicted elimination of Markov violations in the Inhibitory conditions was observed.

## 4. Methods

### 4.1. Materials

Three content domains were tested: economics, meteorology, and sociology. The subjects were first told that the domain they were about to study included four binary variables. In the domain of economics, the variables were interest rates (either low or normal), trade deficits (small or normal), retirement savings (high or normal), and job mobility (low or normal). In the domain of meteorology, the variables were ozone level, air pressure, humidity, and wind drafts. In sociology, they were degree of urbanization, interest in religion, socio-economic mobility, and interest in sports.

The subjects were then presented with a verbal description of the four causal relations that formed the structures in Figure 3. In the Generative condition (Figure 3A), all causal relations were described as generative. For example, one of the generative relationships in the domain of economics was “Low interest rates cause small trade deficits. The low cost of borrowing money leads businesses to invest in the latest manufacturing technologies, and the resulting low-cost products are exported around the world”. In contrast, in the Y_A_-Inhibitory (Figure 3B) and Y_B_-Inhibitory (Figure 3C) conditions, $Y_{A}\to Z$ and $Y_{B}\to Z$ were described as inhibitory, respectively. For example, “High retirement savings prevent high job mobility. With their financial future assured, workers are less motivated to look for new, higher-paying jobs”.

The subjects were then presented with the inference test. Each trial presented the values of one or two variables and asked the subject to predict the state of another. For example, a subject might be told that an economy had low interest rates and a normal trade deficit and be asked the probability of it having a high level of retirement savings. The subjects entered their response by moving a tick on a rating scale with ends labeled 0% and 100%. To ensure that the subjects did not have to rely on their memory, the causal relationships were repeated on the bottom half of the screen. The subjects were presented with the 32 distinct conditional probability queries, including those required to assess the independence of $Y_{A}$ and $Y_{B}$. The order of these queries was randomized for each participant.

### 4.2. Design and Participants

The experiment consisted of a 3 (content domain: economics, meteorology, or sociology) × 2 (Generative versus Inhibitory) × 2 (Y_A_-Inhibitory vs. Y_B_-Inhibitory) between-subject design. The subjects were randomly assigned to these 3 × 2 × 2 = 12 between-subject cells, subject to the constraint that an approximately equal number appeared in each cell. For participating, 112 New York University undergraduates received course credit. This sample size is similar to those in previous tests of causal reasoning using these materials (e.g., Rehder, 2024 [15]).

## 5. Results

**Figure 4:**
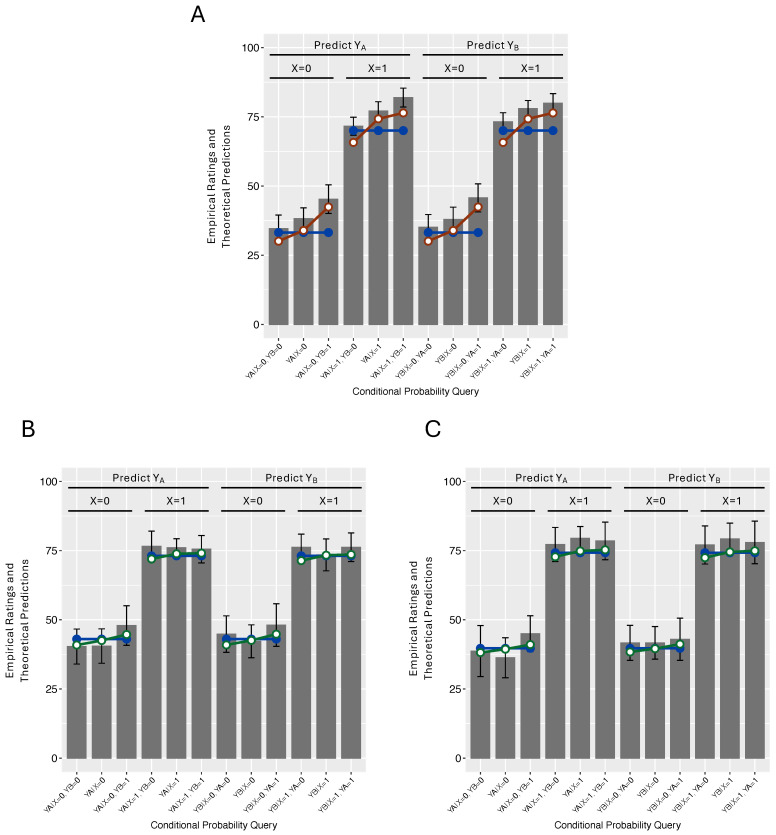
Empirical ratings and theoretical model fits. Panel (**A**): Generative condition. Panel (**B**): Y_A_-Inhibitory condition. Panel (**C**): Y_B_-Inhibitory. The blue lines with closed plot points are the fits of the normative model, the red lines are those of the mutation sampler with 1111 and 0000 prototypes, and the green lines are those of the mutation sampler assuming no prototypes. The error bars are standard errors.

The initial analyses revealed no effect of content domain and so the key inference ratings in each condition are presented in Figure 4 collapsed over that factor. Figure 4A presents the results in the Generative condition. Moving from left to right, the first three bars present the probability of $Y_{A}$ as a function of whether $Y_{B}$ is absent, unknown, or present, given that *X* is absent. The next three bars are the same queries with *X* present. The final six bars are the same queries with the role of $Y_{A}$ and $Y_{B}$ reversed.

Starting with the Generative condition, Figure 4A indicates that the subjects in this condition committed positive Markov violations: within each triplet of ratings in the figure, the rated probability of one *Y* was lower when the other *Y* was absent and higher when the other *Y* was present. This is a novel result, insofar as, to my knowledge, there have been no prior demonstrations of Markov violations with the causal structure in Figure 4A. Nevertheless, they are unsurprising in light of the widespread presence of positive Markov violations with causal structures with only generative relations (e.g., in Figure 1A,B and Figure 2A).

The more interesting results appear in Figure 4B,C, which show that the Markov violations that were so prominent in Figure 4A are largely absent in the Y_A_-Inhibitory and Y_B_-Inhibitory conditions. Although these results are surprising, in light of the demonstrations of pervasive Markov violations reviewed above, recall that they were predicted on the basis of the mutations sampler’s assumption regarding the absence of prototype states in these conditions.

**Figure 5:**
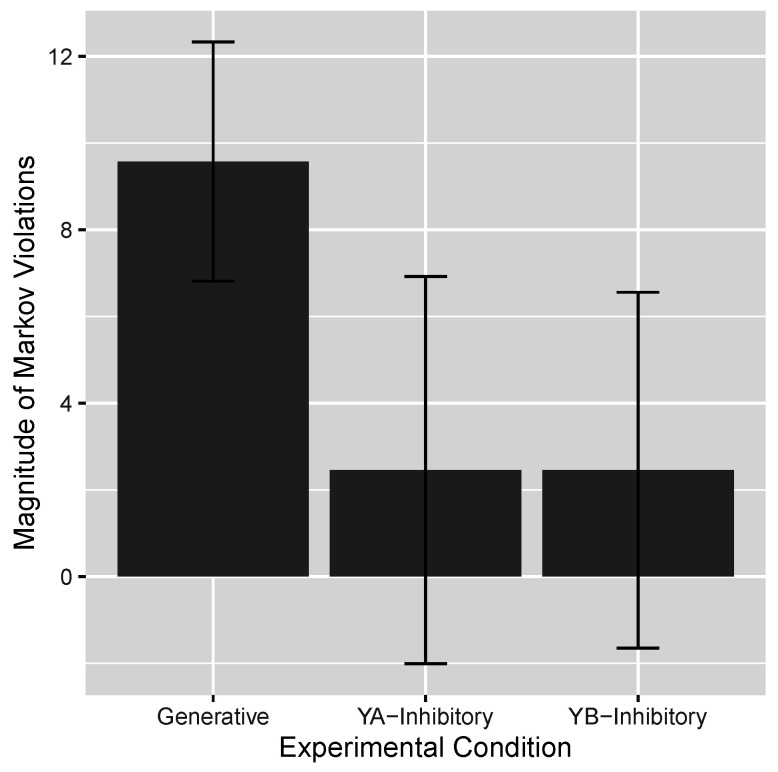
The magnitude of the Markov violation in each condition. Generative condition. Y_A_-Inhibitory condition. Y_B_-Inhibitory condition. The error bars are 95% confidence intervals.

A summary measure of the Markov violation in the three conditions was computed by subtracting $p(Y_{i}=1|C,Y_{j}=1)$ from $p(Y_{i}=1|C,Y_{j}=0)$ for each pairing of the Ys and each level of *C* and averaging the results. These results are presented in Figure 5. The average Markov violation in the Generative condition was 9.6, a result quite comparable to other studies that tested networks with generative relations. In contrast, in the Y_A_-Inhibitory and Y_B_-Inhibitory conditions those violations were a much more modest 2.5 and 2.5, respectively. A one-way ANOVA of these results yielded a statistically significant effect of condition, F(2,109)=5.77,p = 0.004. Individual *t*-testing of the three conditions yielded a statistically significant Markov violation in the Generative condition, $t\left(55\right)=6.80,p<10^{-8},BF>10^{6}$, but not in the other two conditions, both *p*s > 0.25. and BFs < 0.38 (Bayes factors were computed via the R BayesFactor package, version 0.9.12-4.5.).

### Theoretical Modeling

Two versions of the mutation sampler were fitted to each subject’s 32 conditional probability judgments. One version assumed the prototypes that are deemed appropriate for causal networks with all generative relations; namely, ones where the variables are all present (1111) or all absent (0000). The other version assumed the absence of prototypes, which is appropriate for causal networks for which no qualitatively consistent set of variable values exist, such as those in the Y_A_-Inhibitory and Y_B_-Inhibitory conditions. For this model, sampling began at a randomly chosen system state. For both models, the fitting procedure involved identifying the best fitting values for $w_{X}$ (the probability of the root cause *X*), $w_{Z}$ (the strength of the exogenous causes of *Z*), and λ (the mutation sampler’s chain length parameter). A single parameter $w_{gen}$ estimated the strength of all the generative causal relations. In the Y_A_-Inhibitory and Y_B_-Inhibitory conditions, an additional parameter estimated the strength of the single inhibitory relation ($w_{inh}$). Additional details of the model fitting procedure can be found in [15]. Table 1 presents each model’s best fitting parameters averaged over the participants in each condition, along with a number of measures of fit, including the correlation between predicted and observed values averaged over participants (*R*) and a measure (*AIC*) that takes into account a model’s number of parameters (AIC = n*log (SSE/n) + 2*(p + 1), where *SSE* = sum of squared error, *n* = number of data points fit, and *p* = a model’s number of parameters. Thus, lower values of AIC reflect a better fit. This measure has been deemed appropriate for comparing models fit by least squares [31]. The last column of Table 1 presents the percentage of participants best fitted by that model in each condition.

The results of these fits averaged over subjects are shown in Figure 4 superimposed on the empirical data. As expected, Figure 4A indicates that the mutation sampler with prototypes did a good job reproducing the subjects’ positive Markov violations. Table 1 indicates that these effects resulted in it yielding a better fit (AIC of 166.0) as compared to a mutation sampler without prototypes (168.0). According to AIC, 68% of the subjects were better fitted by a mutation sampler that began sampling at either 1111 or 0000.

In contrast, the mutation sampler without prototypes yielded a better fit in both the Y_A_-Inhibitory (AIC of 178.3 vs. 178.6; number of subject fit best = 59%) and the Y_B_-Inhibitory (AIC of 186.3 vs. 186.6; number of subject fit best = 74%) conditions. Figure 4B,C confirm that this model reproduced the *absence* of substantial Markov violations in these conditions.

It is informative to compare the fitted chain length parameters in the Y_A_-Inhibitory and Y_B_-Inhibitory conditions. For the mutation sampler without prototypes, the average chain length parameter in those conditions was 16.2 and 16.6, respectively, values that were very comparable to those estimated in the Generative condition. But the mutation sampler with prototypes estimated much larger chain length parameters (35.6 and 32.3). The reason for this difference was that in these conditions the prototypes led the model to predict Markov violations that the subjects, in fact, did not commit. Larger chain lengths were estimated, in order to minimize the impact of those prototypes and so allow the model’s predictions to be more in line with subjects’ actual judgments. Rather than assuming that the subjects reasoned with prototypes but then sampled excessively to minimize their influence, the more parsimonious explanation offered here is that the subjects in the Y_A_-Inhibitory and Y_B_-Inhibitory conditions did not have a bias in where they started sampling.

Figure 4 also presents the fits of the normative model in each condition. Unsurprisingly, in the Generative condition the mutation sampler with prototypes yielded a better fit than the normative model (AICs of 166.0 vs. 170.0), because the former model reproduced that condition’s Markov violations whereas the latter did not. In contrast, but still unsurprisingly, in the conditions with an inhibitory causal relation the mutation sampler without prototypes did *not* yield better fits than the normative model (AICs of 178.3 vs. 176.9 in the Y_A_-Inhibitory condition and 186.3 vs. 185.1 in the Y_B_-Inhibitory condition). This is unsurprising, because the mutation sampler without a bias in where sampling starts is simply an approximation of the normative model, which means that it will lose any comparison with the normative model due to its extra chain length parameter λ.

## 6. Discussion

In psychological research, one sign of understanding a phenomenon is knowing how to make the phenomenon disappear. Positive Markov violations are so ubiquitous in the causal reasoning literature that failing to observe them is of theoretical importance. Using a theoretical model—the mutation sampler—as a guide, this study derived the specific conditions under which Markov violations should fail to materialize. In fact, asking subjects to reason with causal structures in which there was no setting of the variables that were qualitatively consistent with that structure was sufficient to eliminate Markov violations. In contrast, when subjects reasoned with a causal structure that was identical, except that one of the four causal relations was generative rather than inhibitory, Markov violations of about the same magnitude as observed in other studies once again revealed themselves.

The prototype states that the mutation sampler posits as the underlying cause for Markov violations (and other causal reasoning errors, such as weak explaining away) are intended to reflect a reasoner’s superficial but cost-effective analysis of a causal network. The mutation sampler makes several claims about that analysis. Firstly, it assumes that the direction of causality is ignored so that, for example, the common cause, chain, and common effect networks with only generative relations shown earlier all have the same prototypes. Secondly, quantitative information, such as the strength of the causal relations, is ignored. Thirdly, there is no consideration of the details of how multiple causal influences combine. However, what the present results suggest is that the analysis that yields prototype states *is* sensitive to whether the causal relations are generative or inhibitory, as indicated by the elimination of Markov violations when just one of four generative causal relations was described as inhibitory. Of course, this conclusion is corroborated by the results described earlier, in which the sign of Markov violations changed from positive to negative when a causal network contained a mix of generative and inhibitory relations (but, unlike in the current study, still allowed the identification of qualitatively consistent prototype states) [15].

It is important to have a balanced perspective on the notion of prototype states. Starting sampling at high probability prototypes states might seem generally unwise, given that they introduce systematic errors into people’s causal inferences. But, as mentioned, it has a benefit, as well, which is that it tends to maximize the overall accuracy of a reasoner’s causal inferences when the cognitive resources available are limited [7]. Under this view, Markov violations are the price one must pay in order to maximize the overall quality of one’s causal inferences.

These current results suggest several new avenues for research. One involves the notion of prototypes themselves. The current formulation of the mutation sampler specifies that prototypes are an “all or none” concept; that is, they are system states that are *completely* (qualitatively) consistent with the causal relations. Because they exhibit no completely consistent states, this definition led to the assumption that the networks in Figure 3B,C have no prototypes and, thus, no bias in where sampling starts. But it would be possible to adopt a more graded notion of prototypes whereby system states are chosen as places to start sampling with a probability that is a function of the *number* of inconsistencies (the number of “broken” causal links) they exhibit. For example, for the Y_A_-Inhibitory network in Figure 3B one might specify that sampling was more likely to commence at state $X=1,Y_{A}=0,Y_{B}=1,Z=1$, which has one broken causal link (namely, $X\to Y_{A}$), than at $X=1,Y_{A}=0,Y_{B}=0,Z=1$, which has three ($X\to Y_{A}$, $X\to Y_{B}$, and $Y_{B}\to Z$). The differences in behavior that this alternative view predicts are likely to be subtle but could be explored in future research (I thank an anonymous reviewer for raising the possibility).

More generally, it is worth noting that as a process model the mutation sampler makes predictions about dependent variables that are often not emphasized in the causal reasoning literature. For example, a recent study by Kolvoort et al. aimed to assess the sources of subjects’ response variability; that is, why an individual might give a different answer to logically equivalent causal queries [14]. The mutation sampler predicts this sort of within-subject variability because the outcome of the stochastic sampling process might differ from one invocation to the next. Indeed, Kolvoort et al. found that the mutation sampler provided a better account of that variability than other sources (motor noise in subjects’ responses, misreading the causal query, etc.). As a cognitive process model, the mutation sampler is, thus, well positioned to provide potential explanations of data related to not just *what* causal inferences humans draw but also how they draw them [32,33,34,35,36].

## Figures and Tables

**Table 1:** Fits of mutation sampler with and without prototypes 0000 and 1111.

	Average Parameter Estimates					Measures of Fit		
Model	$w_{X}$	$w_{\mathrm{gen}}$	$w_{\mathrm{inh}}$	$w_{Z}$	λ	R	AIC	% Subjects
Generative								
With Prototypes	0.649	0.476		0.454	17.5	0.815	166.0	67.9
	(0.028)	(0.033)		(0.028)	(1.09)	(0.016)	(3.17)	
No Prototypes	0.699	0.774		0.344	16.9	0.804	168.0	32.1
	(0.045)	(0.032)		(0.039)	(1.90)	(0.015)	(3.32)	
Y_A_Z-Inhibitory								
With Prototypes	0.612	0.456	−0.338	0.479	35.6	0.708	178.6	41.3
	(0.026)	(0.037)	(0.032)	(0.027)	(1.10)	(0.029)	(3.71)	
No Prototypes	0.736	0.714	−0.395	0.440	16.2	0.718	178.3	58.6
	(0.047)	(0.052)	(0.038)	(0.039)	(1.21)	(0.027)	(3.61)	
Y_B_Z-Inhibitory								
With Prototypes	0.619	0.519	−0.396	0.483	32.3	0.714	186.6	25.9
	(0.040)	(0.052)	(0.038)	(0.039)	(1.13)	(0.038)	(3.13)	
No Prototypes	0.673	0.732	−0.441	0.389	16.6	0.728	186.3	74.1
	(0.061)	(0.052)	(0.071)	(0.042)	(1.24)	(0.031)	(3.13)	

*Note*: “% Subjects” are the percentage of subjects best fitted by the mutation sampler. Because they were not normally distributed, the reported average chain length parameter λ was computed by exponentiating the average of the logarithm of the subjects’ individual chain length parameters. Standard errors are shown in parentheses. AIC = Akaike information criterion.

## Data Availability

Data and study materials from these experiments are available: https://osf.io/35qke/, 8 December 2024.
